# Supplementary material for: An information theoretic treatment of sequence-to-expression modeling
Source: PLoS Comput Biol. 2018 Sep 26;14(9):e1006459. doi: 10.1371/journal.pcbi.1006459 (PMC6175532; doi:10.1371/journal.pcbi.1006459)
Supplement: S1 Appendix — (DOCX) [file pcbi.1006459.s001.docx]

**S1 Appendix**

**Scaling the gene expression level measurement for TFs and target genes**

*Scaling of gene expression:* The thermodynamics-based calculation of the GEMSTAT model is given by the formula $\frac{Z_{on}}{(Z_{on} + Z_{off})}$ (Equation 1 in He et al. [22]). This formula represents the fractional occupancy of the basal transcriptional machinery (BTM), but not the gene expression level. The expression $E$ of a gene in a cell type is merely proportional to this fractional occupancy for that cell type. (*Note:* This is a feature of the Shea-Ackers model, from which the GEMSTAT model was derived. The transcriptional initiation rate is assumed proportional to the BTM occupancy, which then allows us to show that the equilibrium mRNA level must also be proportional to that occupancy.) In other words, GEMSTAT is incapable of modeling absolute expression levels. The modeling framework deals with this by either defining the accuracy of the model (goodness of fit) as the correlation between predicted and true expression levels across different cell types, or by assuming an unknown constant of proportionality that must be multiplied with the predicted expression before computing its deviation from true expression. (In the present work, we used the latter approach.) In either case, the scaling of the gene expression levels before presenting them to the model is irrelevant, and our results will be unchanged if we did not scale the expression values to the range 0-1.

*Scaling of TF expression*: GEMSTAT was designed to handle the variation of *relative* TF concentration levels across cell types (e.g., positions along an embryonic axis), since this is what is commonly available. The biophysics of TF-DNA interactions, on the other hand, needs to be formulated in terms of the absolute TF concentration in a cell. Thus, where the model uses the term $[TF]$ (concentration of TF in cell), we replace it with the term $\nu\left[ TF \right]_{rel}$, where $\left[ TF \right]_{rel}$ is the relative TF concentration in that cell. This leads to a free parameter $\nu$ for each TF, but it also means that the $\left[ TF \right]_{rel}$ values can be input on an arbitrary scale. Thus our “scaling” the TF expression level to a range of 0-1 is not necessary, and is merely a convenience for visualization.

Furthermore, it turns out that this additional free parameter per TF does not ultimately add to the model complexity, for the following reason. The key formula modeling TF-DNA interactions in GEMSTAT is the formula for the “statistical weight” of a site $S$ for a TF, whose optimal site is $S_{max}$, given by: $q\left( S \right)=K\left( S_{max} \right)\nu\left[ TF \right]_{rel}e^{LLR\left( S \right)-LLR(S_{max})}$ (Equation 2 in He et al. [22]). Here, $K\left( S_{max} \right)$is the unknown TF-DNA binding constant for the optimal site $S_{max}$, $\nu\left[ TF \right]_{rel}$ represents the TF concentration, and $LLR(S)$ is the traditional LLR score of site$S$. Note that the TF-DNA binding constant $K\left( S_{max} \right)$ must be made a free parameter for each TF. Note also that the two unknown constants $K\left( S_{max} \right)$and $\nu$ only occur as a product and never separately, so for model inference purposes their product can be treated as a single free parameter. This product $K\left( S_{max} \right) \times\nu$ is in fact the free parameter that we refer to as the ‘DNA binding parameter’ in the paper. Thus, the parameter $\nu$ that scales the relative TF concentration to an absolute level does not cause an additional penalty in terms of model complexity.
