# Supplementary figures and images for: An information theoretic treatment of sequence-to-expression modeling
Source: PLoS Comput Biol. 2018 Sep 26;14(9):e1006459. doi: 10.1371/journal.pcbi.1006459 (PMC6175532; doi:10.1371/journal.pcbi.1006459)

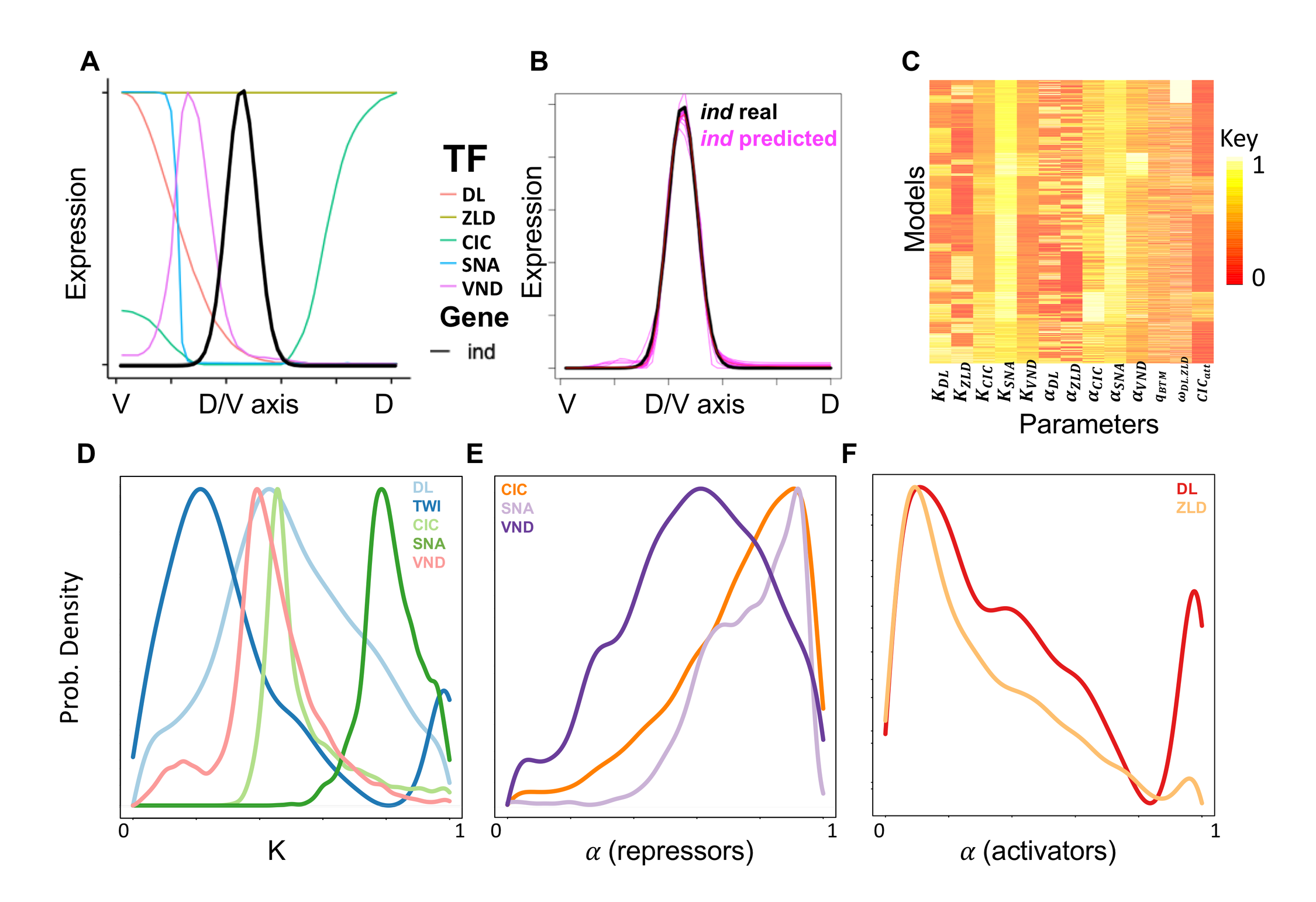

Supplement: S1 Fig — (A) The expression domain for TFs and the ‘ind’ gene is shown along the Dorsal-ventral domain. The x-axis represents ventral (left) to dorsal (right) end of the D/V axis and the y-axis is the expression value from no expression to the maximum observed expression for each gene or TD, on a scale of 0 to 1. (B) Predicted average ind expression (magenta) from all models optimized to fit wild-type data (black). Each pink line shows the prediction of a single model in the ensemble (C) Each row is a model in the ensemble and each column corresponds to a parameter for the model. Each parameter is scaled to the range of 0 to 1. The K parameter for all TFs and α parameter of repressors are in logarithmic scale and the α parameter of activators, cooperativity and qBTM are in linear scale. (D-F) Marginal densities of parameters of the ensemble. Each parameter vector is scaled to be in the same range. The x-axes in (D) and (E) are in logarithmic scale and in F in linear scale. (TIF) [file pcbi.1006459.s002.tif]

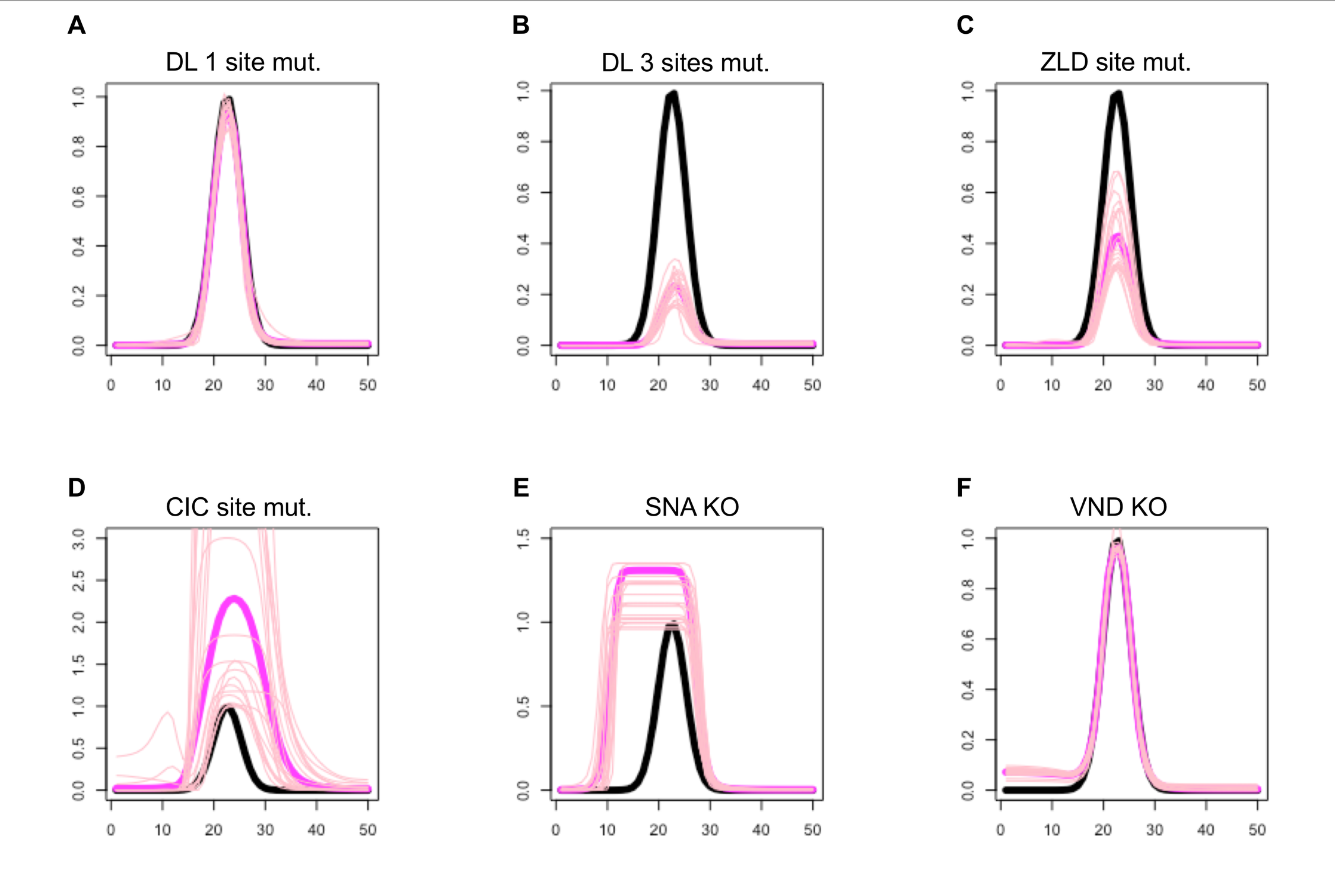

Supplement: S2 Fig — The wild-type ensemble was filtred to retain only models that correctly predict the observed effects of an additional perturbation experiment (S1 Table), thus yielding a smaller, filtered ensemble for each experiment. In each panel, wild-type ind expression is shown in black, pink curves represent predictions of models in the ensemble and magenta represents a weighted average of the ensemble predictions. (A) No change is observed in the expression when the strongest DL site is mutated. (B) Peak ind expression is reduced by 65% after 3 DL sites are mutated. (C) Peak ind expression is reduced by half upon mutations in ZLD binding sites. (D) ind expression expands dorsally when two sites of CIC is mutated. (E) ind expression expands ventrally in VND knockout. (F) The expression of ind is not changed in SNA knockout experiment. (TIF) [file pcbi.1006459.s003.tif]

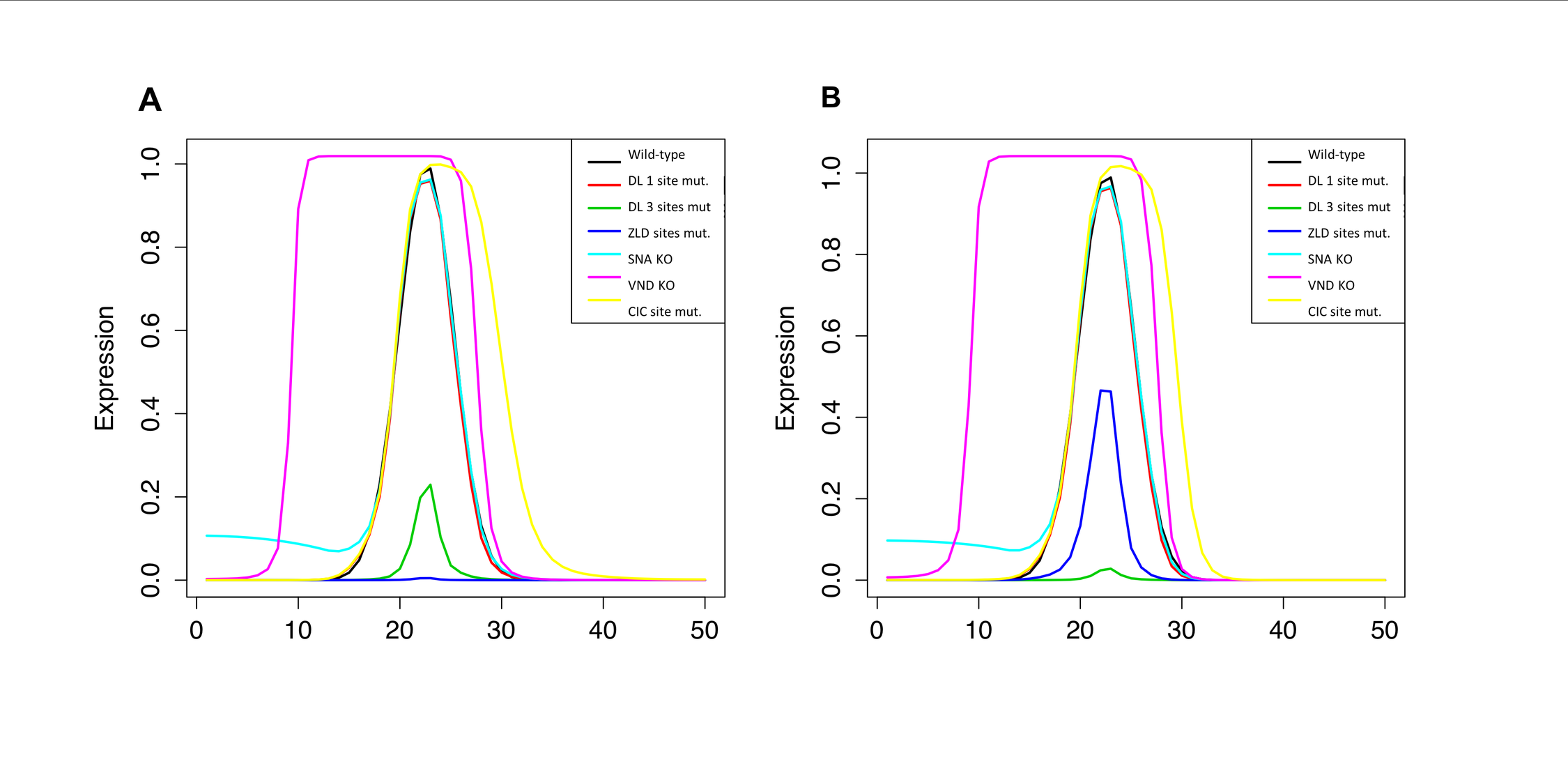

Supplement: S3 Fig — We searched for a model that not only has a good fit to the wild-type ind expression but also produces the known effects of perturbation experiments reported in the literature (S1 Table). Such a model would then be used as the ‘truth’ for predicting the effects of other ‘experiments’. Starting from the wild-type ensemble, we filtered models that predict the effect of CIC site mutation (‘CIC site Mut.’), VND knockout (‘VND KO’), SNA knockout (‘SNA KO’) and mutagenesis of the strongest predicted DL site (‘DL 1 site Mut.’) correctly, resulting in an ensemble of a few hundred models. Then, we checked the ability of these models to reproduce the effect of an experiment where three overlapping DL sites were mutagenized (‘DL 3 site Mut.’) and another experiment where the four strongest ZLD sites were mutagenized (‘ZLD site mutation’). We were unable to find any model that could reproduce both results correctly. Thus, we used only one of these two filters to obtain an ensemble of models that can predict five out of six perturbation experiments correctly. Shown are the predictions for the wild-type condition and the six perturbation conditions, made by two distinct models, both of which fit wild-type data and perturbation experiments ‘CIC site Mut.’, ‘VND KO’, ‘SNA KO’, ‘DL 1 site Mut.’ as well as either (A) ‘DL 3 site Mut.’ (this model fails to reproduce the effect of ‘ZLD site Mut.’) or (B) ‘ZLD site Mut.’ (this model is unable to reproduce the effect of ‘DL 3 site Mut.’). (TIF) [file pcbi.1006459.s004.tif]

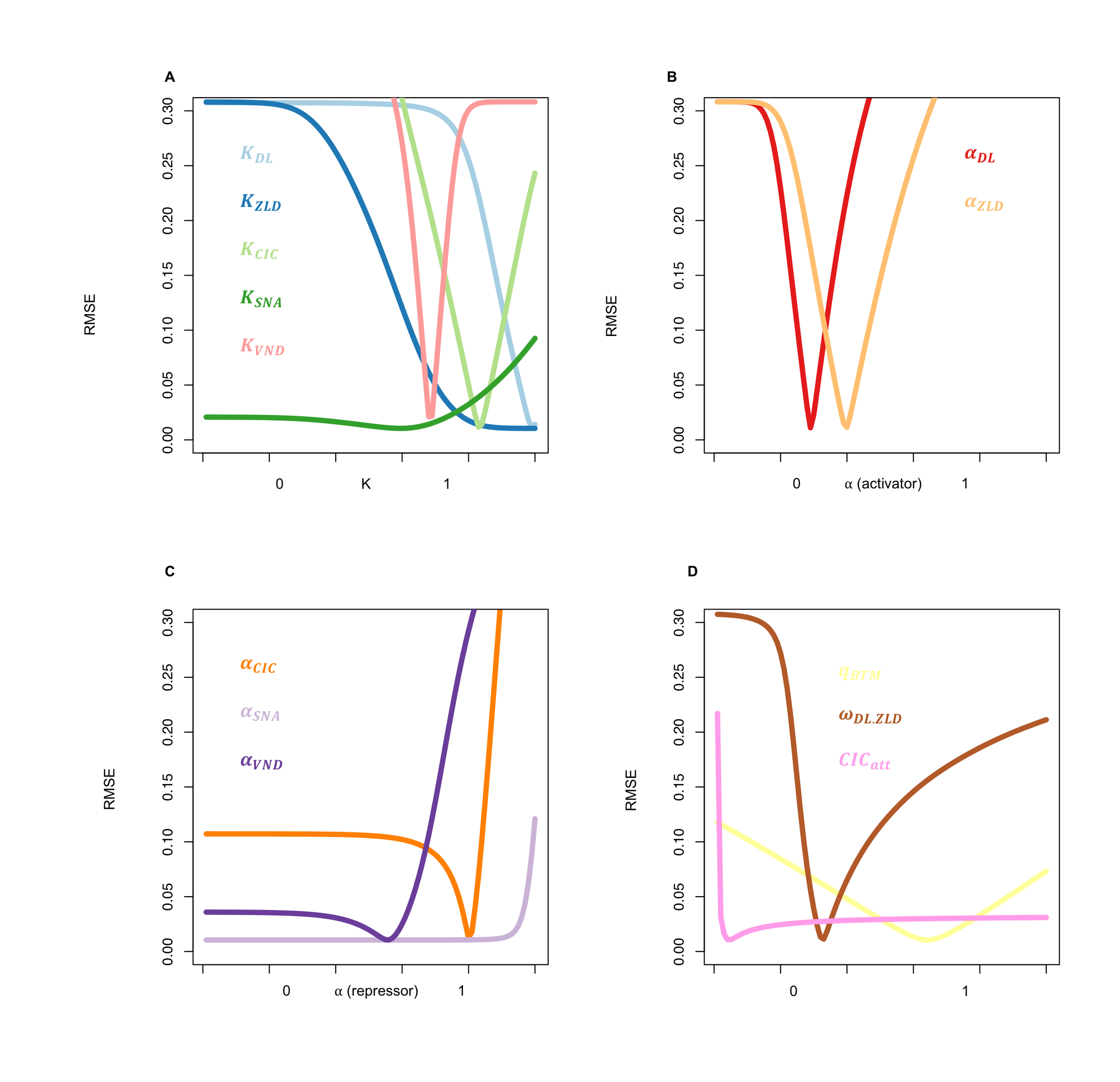

Supplement: S4 Fig — (A-D) Panels show the RMSE scores of the model as the corresponding parameter’s value is varied within its range, keeping other parameters fixed at their optimized values. (TIF) [file pcbi.1006459.s005.tif]

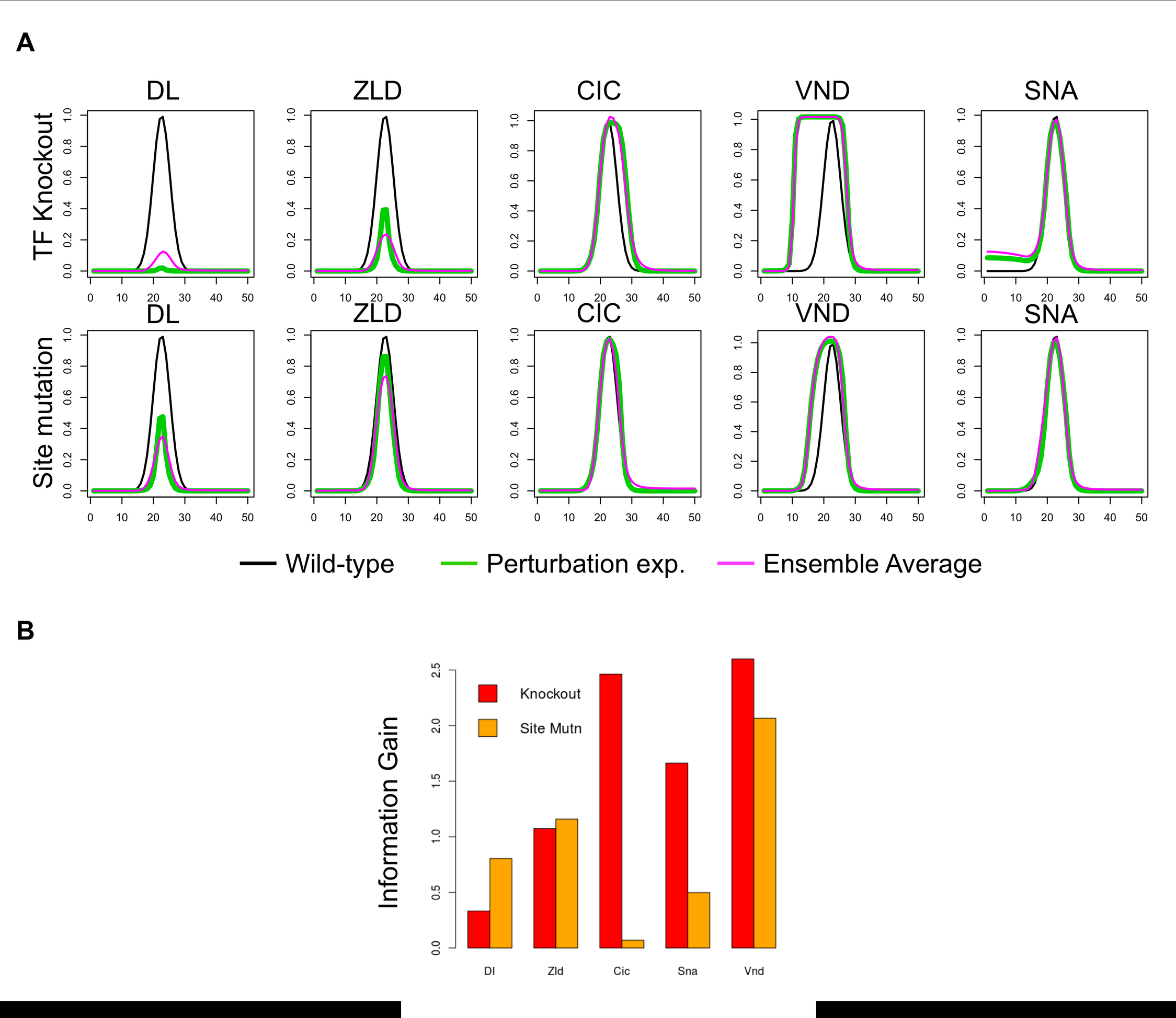

Supplement: S5 Fig — (A) The model is used to generate synthetic ‘experimental’ results of TF knockout (top row) or strongest site mutagenesis (bottom row), for each TF, shown in green. These are compared to the synthetic ‘wild-type’ expression profile of ind, shown in black (in each panel). Magenta curves show the average prediction of the filtered ensemble for each of these ‘experiments’. (B) Information gain due to each synthetic perturbation experiment, with semantics analogous to those in Fig 2B, under the alternative ‘synthetic real’ model MST. (TIF) [file pcbi.1006459.s006.tif]

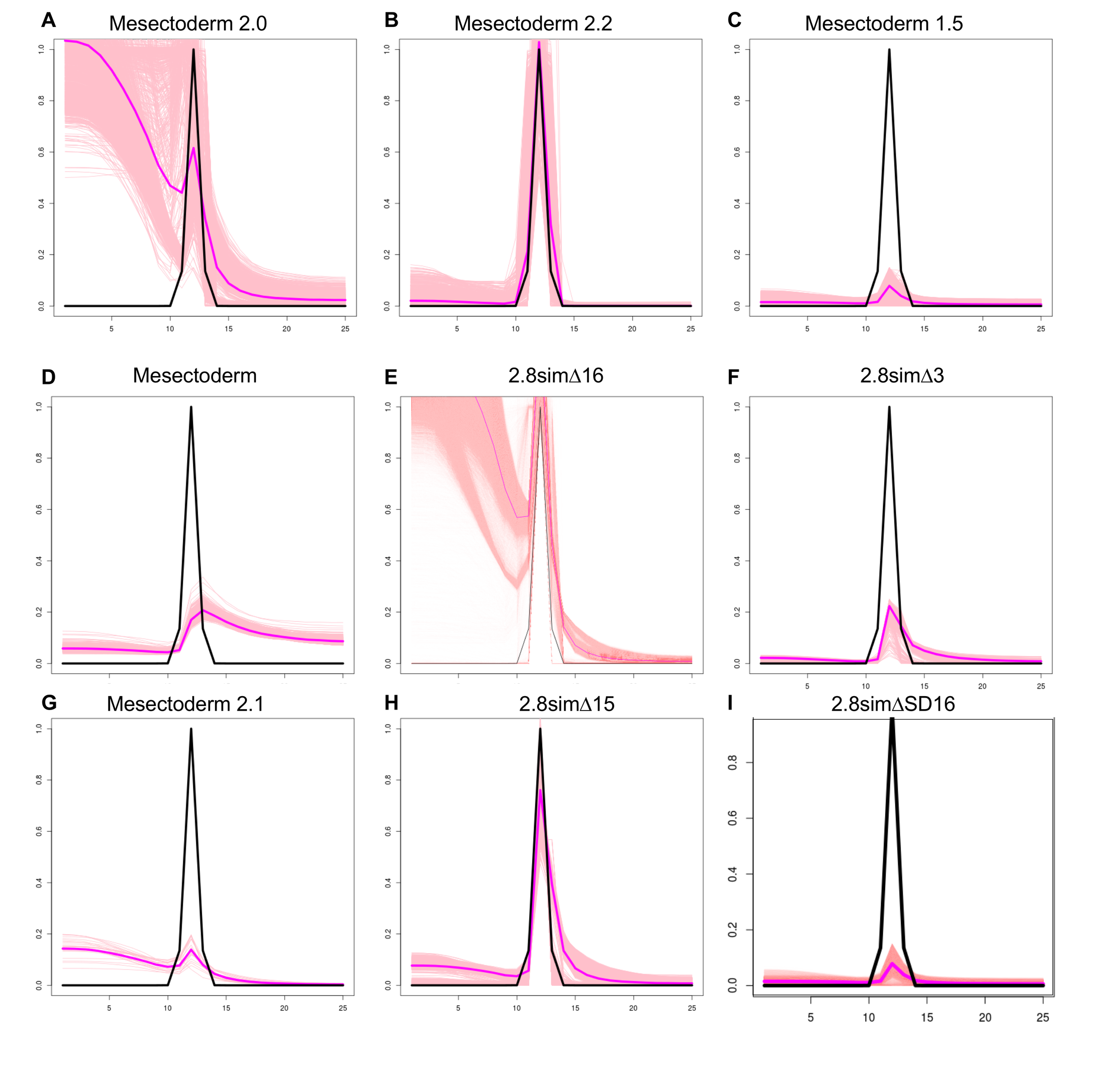

Supplement: S6 Fig — The wild-type ensemble was filtered to retain only models that correctly predict the observed effects of an additional perturbation experiment (S2 Table), thus yielding a smaller, filtered ensemble for each experiment. In each panel, wild-type sim expression is shown in black, pink curves represent predictions of models in the ensemble and magenta represents a weighted average of the ensemble predictions. (A) The expression extends to the presumptive mesoderm (B) No change is observed in the expression when the strongest DL site is mutated. (B) Wild-type expression was observed in mesectoderm. (C) The expression is abolished when using the 1.5 Kb enhancer sequence. (D) The ventral-most line of cells of the neurogenic ectoderm. Weak and variable staining is also detected in more ventral regions of early embryos. (E) Weak expression ("greatly reduced mesectodermal transcription, but a low level of expression was detectable"). (F) Mesectodermal transcription was abolished. (G) No expression is observed. (H) The expression is similar to the wild-type expression. (I) The Expression is completely abolished. (TIF) [file pcbi.1006459.s007.tif]

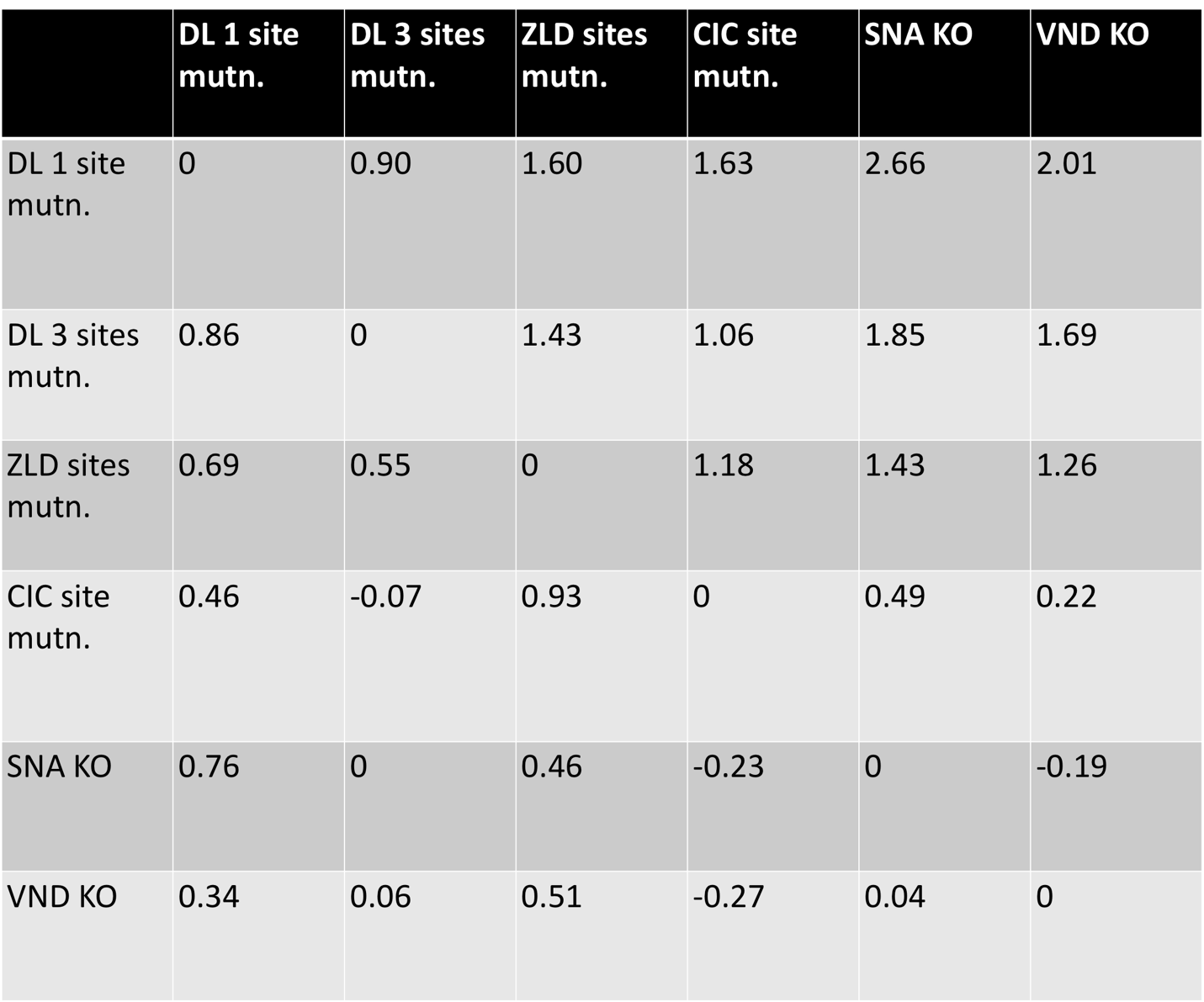

Supplement: S3 Table — The value in row i and column j is the information gain due to experiment j when conducted after experiment i. ‘SNA KO’ and ‘VND KO’ are both informative experiments on their own (Fig 3A), but not when following the other, suggesting that they capture the same type of information. On the other hand, the highest difference between the entropy scores is when the ‘DL 1 site mut.’ experiment is followed by the ‘SNA KO’ experiment (information gain of 2.66). In part this is because the ‘DL 1 site mut.’ experiment on its own has relatively little information gain. (DOCX) [file pcbi.1006459.s010.docx]
