# Supplementary material for: An information theoretic treatment of sequence-to-expression modeling
Source: PLoS Comput Biol. 2018 Sep 26;14(9):e1006459. doi: 10.1371/journal.pcbi.1006459 (PMC6175532; doi:10.1371/journal.pcbi.1006459)
Supplement: S1 Table — For each perturbation experiment reported in the literature, second column summarizes the effect on ind expression and the third column reports a criterion that we selected, based on the observed effect, for determining if a model’s prediction is consistent with that experiment. We used these criteria to filter the wild-type ensemble of models. Note that expression profiles are described with the D/V axis being divided into 50 bins, with the ventral-most position being bin 1 and the dorsal-most position being bin 50. (DOCX) [file pcbi.1006459.s008.docx]

| Experiment  (Source Pubmed ID) | Observation | Filtering Criteria for Model Prediction |
| --- | --- | --- |
| DL 1 site mut. (Strongest site mutagenized.)  (22216201) | No change is observed. | Predicted expression pattern has at most 5% error with SSE measure compared to the same model’s prediction on the wild-type sequence. |
| DL 3 sites mut.  (Three overlapping sites removed.)  (27136354) | Peak expression is reduced by 65%. | Expression is low in all bins outside bins number 22-28 (average expression less than 0.01 of the peak). Peak expression is less than 40% of the wild-type level. |
| ZLD site mut.  (Four strongest sites removed.)  (27136354) | Expression reduced to half of the endogenous levels. | Expression is low in all bins outside bins number 22-28 (average expression less than 0.01 of the peak). Peak expression is less than 60% of the wild-type. |
| CIC site mut.  (Site mutagenized.)  (23733957) | Expression domain expands dorsally, where it matches the spatial domain of the DL protein. | Average expression in bins 40-50 is less than 5% and that in bins 25-35 is greater than 80% of the maximum expression. |
| SNA KO  (SNA knockout.)  (16750631) | No change is observed. | Predicted expression has less than 5% SSE from the same model’s prediction in wild-type conditions. |
| VND KO  (VND knockout.)  (9832511) | Expression expand ventrally, beyond the peak of VND mesoderm region. | Average expression in bins 1-10 is less than 5% of the wild-type and it is more than 80% of the wildtype in bins 20-25. |
