# Supplementary material for: An information theoretic treatment of sequence-to-expression modeling
Source: PLoS Comput Biol. 2018 Sep 26;14(9):e1006459. doi: 10.1371/journal.pcbi.1006459 (PMC6175532; doi:10.1371/journal.pcbi.1006459)
Supplement: S2 Table — For each perturbation experiment reported in the literature, second column summarizes the effect on sim expression and the third column reports a criterion that we selected, based on the observed effect, for determining if a model’s prediction is consistent with that experiment. We used these criteria to filter the wild-type ensemble of models. Note that expression profiles are described with the D/V axis being divided into 50 bins, with the ventral-most position being bin 1 and the dorsal-most position being bin 50. The SSE score is evaluated on the first 25 bins. (DOCX) [file pcbi.1006459.s009.docx]

| Experiment  (Source Pubmed ID) | Observation | Filtering Criteria |
| --- | --- | --- |
| 2.8sim  (9840810) | Wild-type expression is observed in mesectoderm in one row of cells on either side of the embryo. | The peak of expression is at bin 14 and is more than 0.8 in scale of 1. The average expression in bins 13-15 is more than 0.3 and the average expression is less than 1% in all the other bins |
| mesectoderm2.1 (9840810) | No expression | Predicted expression profile differs from a flat line of no expression by an SSE score of less than 5%. |
| mesectoderm1.5 (9840810) | No expression | Predicted expression profile differs from a flat line of no expression by an SSE score of less than 5%. |
| 2.8simΔ3  (9840810) | No expression ("mesectodermal transcription was abolished.") | Peak of predicted expression profile is less than 25% of the wild-type peak. |
| 2.8simΔ16  (9840810) | Weak expression ("greatly reduced mesectodermal transcription, but a low level of expression was detectable") | The average expression in bins 1-9 is more than 50% of the peak in predicted expression profile. |
| 2.8simΔSD16  (9840810) | No expression ("completely abolished mesectodermal transcription") | Predicted expression profile differs from a flat line of no expression by an SSE score of less than 5%. |
| 2.8simΔ15  (9840810) | Mesectoderm ("did not  affect mesectodermal transcription") | Predicted expression profile differs from wild type profile by SSE score of less than 5%. |
| mesectoderm  (15128669) | The ventral-most line of cells of the neurogenic ectoderm. Weak and variable staining is also detected in more ventral regions of early embryos. | Average expression in bins 10-15 is greater than 10% of the peak expression and the average expression in bins 1-9 is less than 5%. |
| mesectoderm2.2  (23101512) | Wild-type expression was observed in mesectoderm. | Predicted expression profile differs from wild type profile by SSE score of less than 5%. |
| mesectoderm2.0  (23101512) | The lacZ expression extends to the presumptive mesoderm | Average expression in bins 1-12 is greater than 80% of the peak expression. The average expression in bins 14-25 is less than 1% of peak expression. |
